# Examination of Patients

**Published:** 1887-08

**Authors:** 


Examination of Patients.—We are all aware that some cases which we are to treat are easy to prescribe for, while others, again, seem to call for no remedy—seem, in fact, to present a chaos of symptoms. Again, in reading our journals some of the cases reported present such a clear picture that we involuntarily name the remedy ; while yet others appear to be so mixed that one cannot decide upon a remedy. Why is this? Does not Hahnemann tell us in his *Organon?* The secret seems to be that the patient in the one case has been thoroughly examined, while in the other the examination has been imperfect.

Hahnemann tells us (§ 104, p. 150): "The totality of the symptoms which characterize a given case, or, in other terms, the image of the disease, being once committed to writing, the most difficult part is accomplished."

Hahnemann here tells us not only to gather the totality of the patient's symptoms, but advises their being put into writing, for he says the physician "can then study it in all its parts, and draw from it all the characteristic marks."

Hahnemann advises us to let the patient and his attendants tell their story in their own way, without interruption or asking leading questions. When the case has thus been narrated the physician asks such questions as may be necessary to render the statement clear or to gain further information. The physician "reads over all that has been communicated to him, and asks of each particular symptom, for example, at what epoch did this or that circumstance occur? Was it previous to the use of the medicines which the patient has taken * * * or while he was taking them, or only a few days after he had discontinued their use? What kind of pain, what particular sensation, was it that was felt in such or such a part of the body? Which precise spot did it occupy? Did the pain come on in separate attacks at intervals, or was it lasting and interrupted? How long did it continue? At what hour of the day or night did it occur?" "Thus the physician causes all the indications which were given in the first instance to be described to him more closely." Never ask a question which indicates the answer, nor one which can be answered by a single yes or no.

If we thus carefully examine our patients there will be found little or no trouble in finding the simillimum. The necessity for close and careful examination of patients is well known to all homœopathic physicians, yet numbers of cases are reported in which the examination has been poorly made. We read numerous cases reported in journals in which no clear indications are given for the remedy selected. Indeed, in many such cases, any one of a dozen remedies is equally called for. When we remember that in some cases the choice between two remedies is decided by one symptom, the necessity for this careful examination which Hahnemann advises becomes apparent.

Thus a case is recalled in which a very careful prescriber had given Arsenic in a case of gangrene when the sufferer would not allow himself to be covered. The doctor overlooked the fact that the Arsenic patient wants to be covered. The Arsenic did no good; a physician was called in consultation, and Secale given with benefit.

Another case occurred which the lamented Hering was treating without success. Finally, after many prescriptions and many failures, the patient said to Hering, "Doctor, I have such a bad spell of coughing every morning about four A. M." Hering exclaimed, "Now I cure you!" And he did.

